# Addressing Hearing Health Care Disparities among Older Adults in a US-Mexico Border Community

**DOI:** 10.3389/fpubh.2016.00169

**Published:** 2016-08-15

**Authors:** Maia Ingram, Nicole Marrone, Daisey Thalia Sanchez, Alicia Sander, Cecilia Navarro, Jill Guernsey de Zapien, Sonia Colina, Frances Harris

**Affiliations:** ^1^Arizona Prevention Research Center, College of Public Health, University of Arizona, Tucson, AZ, USA; ^2^Speech Language and Hearing Sciences, University of Arizona, Tucson, AZ, USA; ^3^Community Health Services, Mariposa Community Health Center, Nogales, AZ, USA; ^4^Department of Spanish and Portuguese, University of Arizona, Tucson, AZ, USA

**Keywords:** community-based participatory research, aging, hearing loss, community health workers, socio-ecological model, health disparities

## Abstract

Hearing loss is associated with cognitive decline and impairment in daily living activities. Access to hearing health care has broad implications for healthy aging of the U.S. population. This qualitative study investigated factors related to the socio-ecological domains of hearing health in a U.S.–Mexico border community experiencing disparities in access to care. A multidisciplinary research team partnered with community health workers (CHWs) from a Federally Qualified Health Center (FQHC) in designing the study. CHWs conducted interviews with people with hearing loss (*n* = 20) and focus groups with their family/friends (*n* = 27) and with members of the community-at-large (*n* = 47). The research team conducted interviews with FQHC providers and staff (*n* = 12). Individuals experienced depression, sadness, and social isolation, as well as frustration and even anger regarding communication. Family members experienced negative impacts of deteriorating communication, but expressed few coping strategies. There was general agreement across data sources that hearing loss was not routinely addressed within primary care and assistive hearing technology was generally unaffordable. Community members described stigma related to hearing loss and a need for greater access to hearing health care and broader community education. Findings confirm the causal sequence of hearing impairment on quality of life aggravated by socioeconomic conditions and lack of access to hearing health care. Hearing loss requires a comprehensive and innovative public health response across the socio-ecological framework that includes both individual communication intervention and greater access to hearing health resources. CHWs can be effective in tailoring intervention strategies to community characteristics.

## Introduction

Across the world people are living longer, increasingly challenging countries to address the cumulative and chronic disabilities that accompany old age (1). On a global level, hearing loss is the most prevalent disabling condition; however, lack of trained hearing health professionals and limited access to assistive technology restricts access to hearing health-care services (2). In the U.S., where the population is rapidly aging, hearing health is only recently emerging as a public health concern (3). Long considered a regrettable, but inevitable manifestation of advanced years, there is growing awareness of the broad implications of hearing loss. Age-related hearing loss is associated with impairment in daily physical activities (4) and increased risk of falls, possibly related to the negative impact of hearing impairment on balance or awareness of surrounding environments (5). The impact of hearing loss on quality of life is well documented (6), but more recent studies have demonstrated an association between hearing loss and accelerated cognitive decline (7) and even dementia (8). The collective impact of these sequelae can subsequently affect the ability to live independently (3). The prevalence of hearing loss coincides with the aging process; 15% of people between 50 and 59 experience hearing loss compared with 63% of those between the ages of 70 and 79 years (3). While hearing aids can ameliorate the negative impact of hearing loss on quality of life indicators (9), only a fraction of adults in the U.S. purchase hearing aids and use them regularly (3). With the expanding need for hearing health care being met only marginally for those who can afford assistive hearing technology, a comprehensive public response is long overdue (10).

Along the U.S.–Mexico border, social and economic conditions contribute to profound disparities in hearing health care comparable to those experienced by developing countries. Mexican Americans with hearing loss are significantly less likely to wear hearing aids compared with Whites (11). In this paper, we present the results of a qualitative study of hearing loss in Nogales, Arizona that draws from a diversity of perspectives to explore the multiple influences on hearing health and access to hearing health care. The purpose of the study is to use findings to develop an integrated public health approach that incorporates the clinical science of audiology within a broad social–ecological framework.

The socio-ecological model (SEM) is an appropriate framework to study hearing health because it moves beyond the cognitive determinants of individual actions to encompass the larger social, cultural, and economic contexts that influence health-related behavior (12, 13). In a border community facing social inequities related to poverty and resource scarcity, the SEM both acknowledges challenges, while identifying and channeling community assets for positive and sustainable community-level change (14). The SEM has provided an equity lens for the development of myriad health promotion efforts over the past 20 years (14–16), including school-based physical activity (17), diabetes (18), and even the use of hearing protection among manufacturing workers (19). However, we found no examples of the SEM addressing hearing loss among older adults. This study explores individual, social, organizational, community, and policy related factors from the perspective of those directly impacted, their family and friends, the health-care system, and the broader community.

## Materials and Methods

The study utilized a community-based participatory approach (CBPR) that combined local expertise of a federally qualified health center (FQHC), funded by the U.S. government to meet the health needs of vulnerable populations, with research faculty from departments of audiology, public health, and Spanish. CBPR ensures that research questions are culturally appropriate, enhances data collection, and facilitates translation of research findings into social change (20–22). FQHC community health workers (CHWs) who function as liaisons between the clinic and the community to increase access to services and quality culturally competent care (23, 24) were central to the participatory process. CHWs are increasingly involved in improving the quality of health research by improving participant recruitment and retention (25). As cultural brokers for the community (26), the CHWs were engaged in development of the research protocols and questions, facilitated interviews and focus groups, and participated in collaborative discussions regarding potential intervention. Partners developed research instruments in either English or Spanish, and translated questions using the concept of functional adequacy of the translation (in addition to the meaning of the text) rather than a direct translation. As an alternative to back-translation, this approach can provide high quality translations in cross-cultural research (27).

### Data Collection

Santa Cruz, County is a U.S.–Mexico border county with a population of approximately 46,000 residents. The FQHC, founded in 1980, is the largest health-care provider in the county, serving approximately 21,000 patients, the majority of whom are Hispanic (90%). Given that the focal point of our study was access to hearing health care, it was advantageous that the partnership included the FQHC where the majority of residents who are uninsured or on public insurance receive primary care services. Data collection methods were based on a convenience sample and corresponded to the SEM domains displayed in Table 1. In March 2014, the CHWs facilitated five community-level focus groups, recruiting participants from the clinic and their health promotion programs. In June 2014, the partnership organized two community hearing screenings, advertising the event in the clinic and in community settings. The CHWs subsequently invited people identified with hearing loss to participate in interviews. The CHWs asked participants to identify family members interested in participating in separate focus groups that explored the nature of communication with people with hearing loss. While not all of those invited to an initial focus group showed up at the appointed time, of those who did come all agreed to participate. Additionally, all of those individuals who were invited by their family members with hearing loss to participate in the family focus groups also agreed to participate. On the organizational level, audiology faculty interviewed primary care providers and their clinical teams at the FQHC, two otolaryngologists and an audiologist who provide iterant service in the community, and the only Nogales hearing aid dispenser.

**Table 1:** **Data collection activities related to socio-ecological model domains**.

Intrapersonal: HOH^a^ older adults	Interpersonal: family/friends	Organization: FQHC providers	Community: Nogales	Community: Nogales
Interviews (*n* = 20)	Focus groups (*n* = 27)	Interviews (*n* = 12)	Focus groups (*n* = 47)	Hearing screening (*n* = 100)
CHWs recruited HOH individuals over 50 years of age from hearing screenings. CHWs conducted 20 interviews that explored the experience of living with hearing loss, perceived response of family members, community resources and access to care.	CHWs invited family members of HOH individuals who participated in interviews to focus groups. CHWs led three focus groups regarding experience as a communication partner to someone with hearing loss, coping strategies, and efforts to access hearing health care.	FQHC staff contacted primary care providers, medical assistants and local hearing aid dispenser. Audiology faculty interviewed 12 providers on perspectives on hearing loss among patients, standards of care, perceptions of patient and family response to hearing health issues and access to care.	CHWs recruited individuals from clinic and health promotion programs. CHW-led 5 focus groups with 8–10 participants regarding perceptions and experiences of hearing loss, community resources and access to care.	CHWs advertised free screenings at the clinic and in the community. Audiology faculty conducted 2 full-day hearing loss screenings and made referrals to primary care doctor for follow up.

*^a^Hard of hearing*.

Community health workers are increasingly involved in improving the quality of health research by improving participant recruitment and retention (25). The CHWs had over 10 years of experience in group facilitation and had worked on previous research efforts. The public health partners facilitated additional interactive workshops to further develop their focus group and interview skills. All participants gave written informed consent under approval from the University of Arizona Human Subjects Internal Review Board. We recorded and transcribed the focus groups and interviews, which were in Spanish with the exception of the provider interviews which were in English.

### Data Analysis

One team member from both audiology and public health conducted content analysis using the SEM domains as a coding guide (28). Analysis focused on health care in the organizational domain, while in the community domain we coded for factors of relevance to a population-based intervention. Consistent with qualitative analysis based on consensus, both coders reviewed the data within each domain and recoded any data that we agreed had been mis-categorized (29, 30). The full team reviewed the themes and the corresponding data to ensure that we had captured all perspectives.

## Results

### Intrapersonal Domain

People with hearing loss described various negative emotions in response to hearing loss, including depression, sadness, desperation, frustration, shame, and embarrassment. These feelings were largely related to others’ reactions to their inability to hear.

I think that this has caused problems to my self-esteem, my value as a person, as a woman, in everything that I do.

Well it makes you ashamed because a lot of times they don’t even know what to say… sometimes I answer and they’re staring at me and I start wondering what I said, and I feel bad.

Hearing loss directly impacted their engagement in social interaction, and for some affected the nature of their personality and self-concept.

Well, I don’t go out much, I don’t socialize much. My outings are with my daughter on an errand, to the pharmacy, to the doctor, but I hardly go out. Especially because of this problem (hearing loss). It has isolated me and repressed me emotionally, because before I was very social. And all this is getting worse.

While several respondents said that they were learning to live with hearing loss, others were motivated to seek assistance.

It motivates me, needing to hear better. I was always very active, I worked for years. When I realized I was losing my hearing, it made me- even though I was discouraged and had low self-esteem, it gave me the strength to want to do something.

Interview participants were well aware of the scope of the impact of hearing loss on their relationships with their family members.

It makes me feel desperate with my kids because they are impatient with me. I tell them, “Write down what you want to say to me in a notebook and I’ll answer.” They get mad at me and they make fun of me. It makes me angry, because they say, “You’re deaf!” But how is that my fault?

They treat me well and they understand me. I don’t have any complaints about my family, my daughter, my son-in-law, my grandkids. But it also makes me sad that there is a lot I don’t understand. My daughter says, “Yes, I told you, mom, I told you, but you didn’t hear me.” Well, yeah, I tried but I didn’t hear you.

In this low-resource community, none of the people interviewed had hearing aids, but they described personal strategies for adapting to hearing loss. These included asking people to repeat themselves or speak louder, or for those with better hearing in one ear asking people to address them from one side. Participants admitted to pretending to hear when they didn’t, and many had come to rely on lip reading. Notably, participants rarely mentioned a collaborative strategy that involved family members in improving communication. Rather, hearing loss contributed to a chain of misunderstanding.

I ask them to repeat themselves, sometimes up to three times, and they say, “What’s going on with you?” Or they don’t believe you can’t hear them.

### Interpersonal Domain

Family and friends were cognizant of the social impact that hearing loss had on their loved ones. They agreed that hearing loss caused people to feel isolated and lonely and to suffer from low self-esteem. A notable difference between the interviews and the focus groups was that people with hearing loss overwhelmingly described being sad and ashamed, while family members also spoke extensively about family members being more angry and irritable.

They become rather angry. Their character changes, I imagine it is because of the desperation of not hearing, when before they could hear perfectly. It makes them angry.

This distinction is at the crux of the communication issue in which individuals become isolated and feel increasingly lonely and sad, expressed as anger and frustration in the eyes of their family and friends. However, family members, particularly spouses, talked of their own frustration caused by the negative impact of disintegrating communication. Participants expressed annoyance over loud television and radio, being ignored or misunderstood, and having to maneuver through social situations. One participant captured many of these emotions in her response:

Sometimes I say to him: “You don’t understand what they’re saying to you, because they say one thing and you answer another. People don’t know you can’t hear. When you don’t understand, please don’t answer.” Other people also start to feel embarrassed and they stop talking. It happens so often that the person just starts isolating themselves. Many times I’m talking and he is not hearing me. Sometimes he talks so loudly that everyone outside can hear him. “I can’t control my voice,” he says. There is too much conflict between me and him. This is very painful.

Several focus group participants said that it was not difficult communicating with their family members, yet offered few examples of how they had adapted their communication style beyond repeating themselves or speaking more loudly. A major concern for family members was the desire for hearing aids; however, the financial resources required were an area of distress.

This is how I would put it. I need a car for work and I need a hearing aid for my dad, what can I do? Well I’m going to get the car for work so I can feed my kids and the hearing aid for my dad has to wait until next year.

Another participant described convincing her son to help with a hearing aid by saying the following:

I’ll tell you something. Do you know why your dad is like that? Because he is deaf. He is deaf because he worked so that you could eat, get educated, so that you could go to college. And now it’s time for you to do your part and give a little back of everything he has given you.

### Organizational Domain

In our analysis, we triangulated the perspectives of providers, community members, and people with hearing loss for a comprehensive understanding of the experience of seeking hearing health care. Respondents were in agreement that hearing screening was not part of a primary care visit for an older adult, and that they would not ask or refer for hearing unless the patient made it the purpose of the visit. Few people with hearing loss had addressed it in a primary care visit, however, for various reasons. These included barriers in patient–provider communication (including hearing loss), patients did not feel it was enough of a problem to make it the focal point of a visit, or they knew they could not afford hearing aids. By contrast, providers felt limited in their ability to respond because of lack of equipment or scope of practice.

There’s only so much as a primary care provider we can do as far as figure that stuff out. We can’t do hearing testing here. There are a lot of neurological tests we can’t do here. We’re limited by what exactly we can do here and the equipment we have.

When they were aware of a hearing issue, many providers referred their patients to otolaryngology specialists who come from Tucson. However, for some providers, there was no point in referral due to lack of insurance coverage for hearing aids.

Most of the time, I just don’t even give them a referral because (Medicare) says, oh yeah, we’ll cover your visit to go see this specialist, and you send them there, and then they say, oh, we don’t cover the treatment, so you’re spending money to send them somewhere, then they’re not going to pay for the treatment, which means you’re essentially wasting money.

While, in general, the focus group and interview respondents desired an opportunity to seek medical care for hearing loss, providers expressed skepticism regarding both patient motivation to seek care and the medical system to respond.

The elderly have hearing loss issues and they don’t have a lot of resources. In fact, it’s not covered at all, hearing aids. Nor are hearing aids very good.

I don’t know if there are interventions that would really make a difference. If someone is losing their hearing early on, they usually don’t like the hearing aid, it just increases the noise.

### Community Domain

We sought to investigate three aspects of the community with an eye to identifying factors that could be altered through intervention: awareness of hearing loss, resources, and culture. Community members affirmed the need to build awareness about preventing hearing loss, particularly given that factories with loud, heavy machinery are common workplaces for the population in Nogales.

The factories in Nogales Sonora- my mom worked her whole life in those factories, and I think my mom started losing her hearing at age 60, because my mom had a lot of vitality and she never got sick except for her hearing problem.

Acceptance of hearing loss was also a common thread across focus groups and interviews, designing efforts to reduce stigma for people with hearing loss, particularly by educating others on how to communicate. Suggestions included developing programs to share information about hearing loss and how to prevent it.

It would be good to inform service people, especially those who work behind windows, because sometimes we can’t hear a thing. I always blame the people behind me for making so much noise, but it would be better to instruct the service people that they have to speak more clearly.

Community members also suggested hearing screenings at health fairs or as part of routine medical care, and to educate the community not to accept hearing loss as normal. Several people suggested a media campaign to raise the awareness of family and friends.

With respect to resources, providers focused on the availability of medical services which included otolaryngologists from Tucson and a hearing aid dispenser across from the clinic. Among community members, few were aware of audiologic services. Participants not only mentioned hearing aids but also expressed concern that hearing aids were not effective. Focus group participants knew people who had purchased hearing aids through the mail and were dissatisfied with their quality. Surprisingly, the local hearing aid dispenser was the only one who mentioned accessing hearing aids from Mexico, but said that the majority of his clients were from across the border.

Finally, we coded the data for to cultural references that would be relevant to a community-level effort to address hearing loss. Participants stressed that the lack of resources was instrumental in fostering a sense of normalcy and inevitability around hearing loss.

Since there isn’t any help, any resources, it’s assumed that it’s normal. We need a campaign to open the community’s eyes that it is not normal, that they can get help.

## Discussion

Our qualitative study of factors related to the socio-ecological domains of hearing health found that, although not life-threatening, the absence of hearing health care can potentially convert a modifiable disability into a chronic, deteriorating, and debilitating condition. Although hearing aids can assist with mild-to-moderate hearing loss and cochlear implants may be available for those with more severe impairments (31), not one of our study participants had accessed assistance from an audiologist in determining the appropriate hearing assistive device. Several community focus group participants, however, knew people who were dissatisfied with hearing aids obtained through the mail, which in all likelihood were inappropriate for the individuals’ hearing impairment and communication needs (32, 33). These findings are in line with epidemiologic studies documenting socioeconomic and racial/ethnic disparities in access to hearing health care (3, 11).

Our findings confirm the causal sequence of hearing impairment on quality of life among family members reported in audiology studies (34, 35) but emphasize how these effects are aggravated by socioeconomic conditions that make assistive devices an issue of family sacrifice. On an organizational level, providers had varying levels of awareness regarding the prevalence and impact of hearing loss among their patients. With respect to options for care, however, they agreed that financial constraints severely limited their patients’ options (36). This reality is further apparent on the community level where we found a general response that communication problems related to hearing loss are a natural part of aging; compelling those with hearing loss to self-manage their disability without outside resources. Similar to our findings from the community screenings and qualitative assessments, other studies of economically disadvantaged communities do not access hearing health care. Torre et al. (37) found that about 60% of older Latino-Americans residing in California County in the U.S. who reported having hearing loss had not pursued medical or audiological services for diagnostic testing or intervention (37).

Table 2 summarizes key findings and outlines recommendations for a community-based public health audiology response to hearing loss within an SEM framework. The core of the intervention centers on communication between persons with hearing impairment and their communication partners. We recommend the development of culturally tailored audiologic rehabilitation (AR) groups facilitated by CHWs within the community setting as an effective way to address barriers in access to care and improve quality of life. AR groups have the benefit of framing hearing loss as an issue of shared communication rather than an individual disability (38). AR groups have demonstrated positive impact on the quality of life for people with hearing loss and their communication partners by increasing understanding between family members and providing them with tools to improve communication (39–41). While not a replacement for hearing aids, these groups can facilitate acceptance of the use of devices and access to appropriate care. Thus on the organizational level, it is crucial that the health-care system can appropriately identify and respond to hearing health-care needs. While training in these same communication strategies can improve the quality of a primary care visit, we also recommend systems level changes, such as the implementation of screening protocols for hearing loss and increased capacity of clinics to screen for hearing loss. The provision of hearing aids may not be attainable for all those in need within the current health-care delivery system; however, increased attention to this issue on a community level could lead to a number of positive changes. These include increased use of amplification and assistive technology in community settings such as churches or public meeting spaces and the development of hearing aid programs where low-income individuals can obtain re-conditioned hearing aids or assistive technology free of cost or at a low-cost for services. Corresponding to the direct recommendations of research participants, the CHWs could incorporate hearing loss in their health promotion efforts and spearhead a community-wide campaign to build awareness and reduce stigma regarding hearing loss and the use of assistive technology.

**Table 2:** **Recommendations for a comprehensive public health response to hearing loss**.

Domain	Key findings	Recommended intervention strategies
Individual (intrapersonal)	Social isolation/withdrawal Emotional distress Denial of hearing loss Adaptation to improve hearing Challenges such as driving, talking on the phone Language barriers, cost, and distance from specialists affect access to care	Culturally tailored audiologic rehabilitation intervention that focuses on strategies to improve communication for those with hearing loss regardless of access to hearing aids. Self-advocacy training to increase access to audiology services, self-efficacy, and social support for hearing loss.
Family/friends (Interpersonal)	Conflict related to hearing loss Concern over changes in personality of person with hearing loss Motivation to seek help Proactive and reactive adaptation to hearing loss Gathering collective resources to seek care, address hearing loss	Culturally tailored audiological rehabilitation intervention for communication partners who stress mutual responsibility for strategies to reduce conflict and improve communication-related quality of life. Develop support systems for family members.
Health-care provider(organization)	Hearing screening not part of regular care. Challenges in patient–provider communication. Lack of provider expertise and equipment. Low interest and delays in referring patients for hearing tests when patient cannot afford out-of-pocket expense of intervention. Lack of awareness of hearing interventions and rehabilitation beyond hearing aids.	Provide training to medical staff in communication strategies. Develop screening protocols for hearing loss. Provide amplification technology (pocket talkers) for medical visits. Increase FQHC capacity to conduct hearing tests and support self-management of hearing and communication health. Create continuity of care in hearing health care.
Community (Nogales)	Acceptance of the problems of hearing loss as part of aging. Lack of hearing health resources. Lack of peer support for hearing loss and its management. Need for community-level information.	Train FQHC CHWs to recognize hearing loss and include communication strategies into health promotion efforts. Advocate for the increased use of assistive listening devices in community settings. Implement a community campaign about solutions to living with hearing loss. Develop a used hearing aid bank to increase hearing aids and recruit audiologists/dispensers to provide fitting services.
Policy	Medicaid/Medicare does not cover hearing aids or rehabilitative services for adults in Arizona. Hearing and other communication disorders not monitored within state-wide surveillance systems.	Improve access to hearing health care for underserved populations including minority and rural older adults. Include hearing health within state-level public health surveillance.

Finally, a sustainable policy response is needed to address the prohibitive cost of hearing assistive technology. Recognizing hearing loss as a public health issue is surely a first step in that process (42). Toward that end, project partners advocated for the inclusion of a hearing health question on a U.S. cross-sectional survey, the Behavioral Risk Factor Surveillance System, in the state of Arizona. The decision of the State Health Department to include the question on a state-level, as well as provide accommodations for individuals with hearing loss to participate in the survey, marked a systems level victory. Survey results will potentially elevate the awareness of hearing health in a state with an aging population (43), and contribute to policy change related to hearing health care.

## Conclusion

Our research challenges assumptions that in the absence of an integrated health system that includes hearing health, affected individuals, and their families can effectively manage hearing loss. This qualitative study in a low-resource Mexican American community makes clear the encroaching impact of hearing loss on healthy aging, and underscores the importance of comprehensive and innovative public health approaches across the socio-ecological framework. The engagement of CHWs in assessing and addressing hearing health in communities that suffer from hearing health disparities can be an effective way to tailor intervention strategies to community characteristics.

## Declaration of Ethical Review

This research was conducted in accordance with the University of Arizona Human Subjects Institutional Review Board.

## Author Contributions

MI was responsible for overseeing qualitative data collection and analysis, collaborative development of data collection instruments, and development of the manuscript. NM was responsible for overall conceptualization of the research design, collaborative development of data collection instruments, and contributed significantly in the writing the manuscript. DS was responsible for collaborative development of data collection instruments, assisting in data collection and analysis, and contributed to the writing of the manuscript. AS was responsible for collaborative development of data collection instruments, data collection, and revising the final manuscript. CN was responsible for collaborative development of data collection instruments, data collection, and contributed to revising the final manuscript. JZ was responsible for collaborative development of data collection instruments, contributed to conceptualization of the qualitative research design, and assisted in the development of the manuscript. SC contributed to the cultural relevancy of qualitative research instruments and provided input into development of the manuscript. FH contributed to overall conceptualization of the research design, collaborative development of data collection instruments, and contributed significantly to the writing of the manuscript.

## Conflict of Interest Statement

The authors declare that the research was conducted in the absence of any commercial or financial relationships that could be construed as a potential conflict of interest.
